# A SNP-based phylogenetic analysis of *Corynebacterium diphtheriae* in Malaysia

**DOI:** 10.1186/s13104-018-3868-6

**Published:** 2018-10-25

**Authors:** Shirley Yi Fen Hii, Norazah Ahmad, Rohaidah Hashim, Yii Ling Liow, Muhammad Adib Abd Wahab, Mohd Khairul Nizam Mohd Khalid

**Affiliations:** 1 Bacteriology Unit, Infectious Diseases Research Centre, Institute for Medical Research, Jalan Pahang, 50588 Kuala Lumpur, Wilayah Persekutuan Kuala Lumpur Malaysia; 2 Molecular Diagnostics and Protein Unit, Specialised Diagnostics Centre, Institute for Medical Research, Jalan Pahang, Kuala Lumpur, 50588 Wilayah Persekutuan Kuala Lumpur Malaysia

**Keywords:** Diphtheria, Malaysia, Toxigenic, Pan-genome analysis, Phylogenetic tree

## Abstract

**Objective:**

There is a lack of study in *Corynebacterium diphtheriae* isolates in Malaysia. The alarming surge of cases in year 2016 lead us to evaluate the local clinical *C. diphtheriae* strains in Malaysia. We conducted single nucleotide polymorphism phylogenetic analysis on the core and pan-genome as well as toxin and diphtheria toxin repressor (*DtxR*) genes of Malaysian *C. diphtheriae* isolates from the year 1986–2016.

**Results:**

The comparison between core and pan-genomic comparison showed variation in the distribution of *C. diphtheriae*. The local isolates portrayed a heterogenous trait and a close relationship between Malaysia’s and Belarus’s, Africa’s and India’s strains were observed. A toxigenic *C. diphtheriae* clone was noted to be circulating in the Malaysian population for nearly 30 years and from our study, the non-toxigenic and toxigenic *C. diphtheriae* strains can be differentiated significantly into two large clusters, A and B respectively. Analysis against vaccine strain, PW8 portrayed that the amino acid composition of toxin and DtxR in Malaysia’s local strains are well-conserved and there was no functional defect noted. Hence, the change in efficacy of the currently used toxoid vaccine is unlikely to occur.

## Introduction

Corynebacterium *diphtheriae* is the causative agent for diphtheria, an acute, communicable disease among children, which can be fatal. The disease is transmitted through contact with respiratory droplets from infected individuals. During the pre-immunization era, diphtheria toxin (tox) was the major cause of mortality in the infected individuals. The disease showed a tremendous reduction after the introduction of toxoid vaccine (PW8) in the twentieth century and currently remaining at less than 8000 reported cases worldwide in year 2016 [1]. The clinical presentation is generally characterized by the formation of an inflammatory pseudomembrane at the upper respiratory tract [2]. The interaction between the bacteria and its infecting phage plays an important role in the bacterial toxin acquisition. DtxR, an iron-dependent toxin repressor produced by *C. diphtheriae* regulates the expression of *tox* introduced via corynebacteriophage by repressing the transcription of *tox* under high iron condition and vice versa [3, 4].

In Malaysia, diphtheria toxoid vaccine is listed in the Malaysia immunization schedule and provided by the Ministry of Health Malaysia. However, not all parents bring their children for vaccination as it is not mandatory. The unvaccinated individuals would be at high risk to acquire the disease from potential diphtheria carriers. Sporadic cases were spotted over the years and recently, there was a sudden surge of diphtheria cases in year 2016, with 31 cases compared to 4, 2, 4 cases in year 2013, 2014 and 2015 respectively [5]. Our study provides the general overview of Malaysia’s *C. diphtheriae* by determining the relatedness among local *C. diphtheriae* isolated within 31 years (from year 1986 to 2016) and comparing these strains with other strains worldwide using single nucleotide polymorphism (SNP) analysis. We also studied the genetic variability of *tox* and *DtxR* in these strains.

## Main text

### Materials and methods

A total of eighty *C. diphtheriae* isolates comprising of 58 toxigenic and 22 non-toxigenic strains from Malaysia, India, Belarus, Africa, Brazil, United Kingdom, Italy and USA were analysed in this study including 28 Malaysia’s isolates (27 toxigenic and 1 non-toxigenic) which we had submitted previously to GenBank under project PRJNA345527 [6]. All the 27 toxigenic strains showed positive Elek test [7]. The other selected genomes were selected randomly from the *C. diphtheriae* strains deposited in GenBank [8–11]. All the genome data used in this study and their accession numbers were specified in Table 1. The construction of the phylogenetic trees was done using kSNP version 3.0 [12] at k-mer = 19 and illustrated by FigTree version 1.4.3 [13]. Two individual phylogenetic trees were constructed based on the SNPs in core genome and pan genome. For pan genome analysis, only the shared SNPs found in at least 90% of the genome were considered. The change of the SNPs is inferred by the branch length. The phylogenetic tree was analyzed at bootstrap value > 0.9 and arranged in decreasing order. Multiple sequence alignments for *tox* and *DtxR* genes were constructed and analysed by Clustal Omega [14].

**Table 1:** Designation of the *Corynebacterium diphtheriae* isolate used in the analysis in this study

No.	Culture no./collection no	Year of collection	Origin of strain	GenBank assembly id.
1	C111	1986	Malaysia	GCA_002115305.1
2	C110	1986	Malaysia	GCA_001832975.1
3	C113	1986	Malaysia	GCA_002115335.1
4	C122	1987	Malaysia	GCA_001832945.1
5	C123	1987	Malaysia	GCA_001832925.1
6	C198	n/a	Malaysia	GCA_002213115.1
7	C325	2003	Malaysia	GCA_001832935.1
8	C488	2008	Malaysia	GCA_001833005.2
9	C517	2010	Malaysia	GCA_001833025.1
10	RZ252	2016	Malaysia	GCA_001889785.1
11	RZ319	2016	Malaysia	GCA_001889845.1
12	RZ356	2016	Malaysia	GCA_001889775.1
13	RZ358	2016	Malaysia	GCA_001889855.1
14	RZ373	2016	Malaysia	GCA_001889825.1
15	RZ378	2016	Malaysia	GCA_001889835.1
16	RZ379	2016	Malaysia	GCA_001889765.1
17	C319	2001	Malaysia	GCA_002115215.1
18	C324	2003	Malaysia	GCA_002115285.1
19	C326	2003	Malaysia	GCA_002213075.1
20	RZ553	2016	Malaysia	GCA_002115325.1
21	RZ597	2016	Malaysia	GCA_002115185.1
22	RZ600	2016	Malaysia	GCA_002115155.1
23	RZ632	2016	Malaysia	GCA_002115145.1
24	RZ656	2016	Malaysia	GCA_002115125.1
25	RZ658	2016	Malaysia	GCA_002115085.1
26	RZ659	2016	Malaysia	GCA_002213145.1
27	RZ693	2016	Malaysia	GCA_002115095.1
28	TH1526	2016	India	GCA_001723445.1
29	TH510	2016	India	GCA_001723465.1
30	TH1141	2016	India	GCA_001723455.1
31	TH2031	2016	India	GCA_001742095.1
32	TH1337	2016	India	GCA_001742085.1
33	PW8	1896	USA	CP003216.1
34	NCTC13129	1997	UK	BX248353.1
35	*C7β* ^*tox*+^	1954	–	CP003210.1
36	CDCE8392	–	–	CP003211.1
37	CD31A	1978	Brazil	CP003206.1
38	CD176	1996	Belarus	GCA_002203315.1
39	CD72	1996	Belarus	GCA_002202335.1
40	CD847	1997	Belarus	GCA_002203335.1
41	CD1360	1999	Belarus	GCA_002202495.1
42	CD2929	2004	Belarus	GCA_002202605.1
43	CD5052	2010	Belarus	GCA_002203535.1
44	CD4568	2007	Belarus	GCA_002202925.1
45	CD4728	2007	Belarus	GCA_002203055.1
46	CD1791	2000	Belarus	GCA_002203155.1
47	CD2173	2001	Belarus	GCA_002203895.1
48	CD2225	2001	Belarus	GCA_002203905.1
49	ST378-KZN-2015-45464	2015	Africa	GCA_001875925.1
50	ST378-KZN-2015-45465	2015	Africa	GCA_001875945.1
51	ST378-KZN-2015-45786	2015	Africa	GCA_001875965.1
52	ST378-KZN-2016-48412	2016	Africa	GCA_001876295.1
53	ST378-KZN-2015-45902	2015	Africa	GCA_001876245.1
54	ST378-KZN-2015-45903	2015	Africa	GCA_001876165.1
55	CD50	1996	Belarus	GCA_002201145.1
56	CD1050	1998	Belarus	GCA_002201165.1
57	*NCTC5011* ^*#*^	1932	UK	GCA_000263415.1
58	*NC03529* ^*#*^	1931	UK	GCA_000257885.1
59	VA01*	1999	Brazil	CP003217.1
60	CD241*	1981	Brazil	CP003207.1
61	INCA402*	2000	Brazil	CP003208.1
62	HC01*	1993	Brazil	CP003212.1
63	HC02*	1999	Brazil	CP003213.1
64	HC03*	2000	Brazil	CP003214.1
65	HC04*	2003	Brazil	CP003215.1
66	HC07*	2013	Brazil	GCA_000953975.1
67	BH8*	–	Brazil	CP003209.1
68	Aberdeen*	2009	UK	GCA_000455805.1
69	ISS3319*	–	Italy	CP025209.1
70	ISS4060*	1999	Italy	GCA_001026805.1
71	NCTC11397*	1969	USA	LN831026.1
72	ISS4746*	2000	Italy	GCA_001026845.1
73	ISS4749*	2000	Italy	GCA_001188005.1
74	RZ523*	2016	Malaysia	GCA_002115225.1
75	CD4010*	2004	Belarus	GCA_002202815.1
76	CD4288*	2006	Belarus	GCA_002203015.1
77	CD4502*	2006	Belarus	GCA_002203705.1
78	ST395-KZN-2015-45784*	2015	Africa	GCA_001876175.1
79	ST402-SA-1980-2337*	1980	Africa	GCA_001876285.1
80	CD4461*	2006	Belarus	GCA_002201075.1

* Non-toxin bearing *Corynebacterium diphtheriae*

^#^*Corynebacterium diphtheriae* isolate before mass vaccination

### Results and discussion

In this study, we used a total of 80 genomes including toxin and non-toxin bearing *C. diphtheriae* to create an overview of *C. diphtheriae* strains in Malaysia. With the advance in next generation sequencing, we applied whole genome SNP analysis in our study by comparing the SNPs in core genome and pan-genome which includes the full complement of bacterial genes: core genome and dispensable genome [15, 16]. The relationship between specific geographical locations within Malaysia which consist of Peninsular and East Malaysia were not evaluated in the study. We assumed that there were frequent movements of the probable carriers between these two areas which might affect our analysis.

**Fig. 1:**
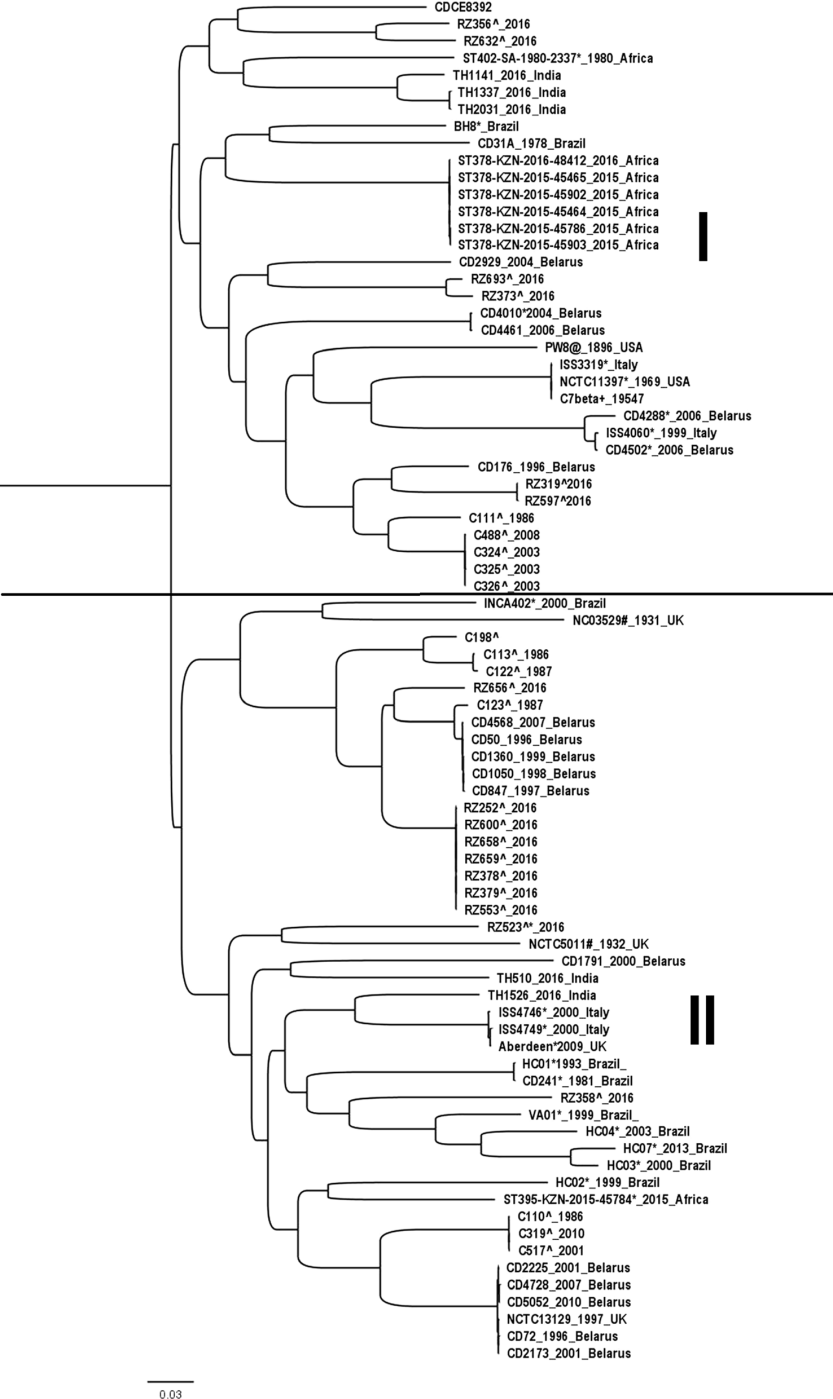
Core genome SNP-based phylogenetic tree analysis of 80 Corynebacterium diphtheriae strains grouped in cluster I and II. The SNPs is only considered if there is at least 90% of the genome has the nucleotide change at the position. ^ and * refer to Malaysia’s and non-toxin bearing isolate, respectively

**Fig. 2:**
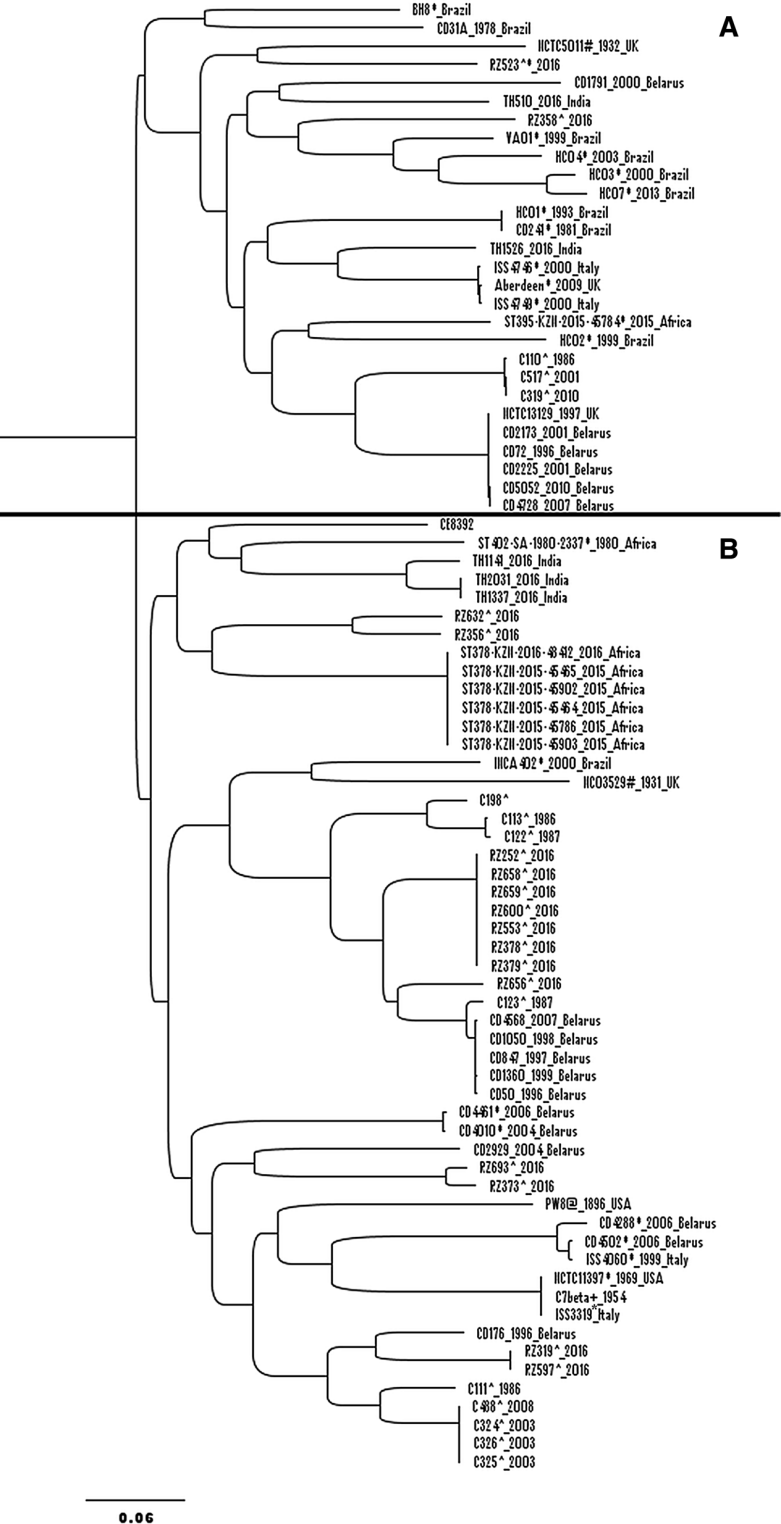
Pan-genome SNP-based phylogenetic tree analysis of 80 *Corynebacterium diphtheriae* strains grouped in cluster A and B. The SNPs is only considered if there is at least 90% of the genome has the nucleotide change at the position. ^ and * refer to Malaysia’s and non-toxin bearing isolate, respectively

Lesser SNPs was observed in core genome (29,184 SNPs) compared to pan-genome (55,071 SNPs). Both core (Fig. 1) and pan-genome (Fig. 2) SNP-based phylogenetic analysis divided the *C. diphtheriae* strains into two large clusters: I, II and A, B respectively. We observed an almost equal percentage of toxigenic and non-toxigenic strains in cluster I and II using core genome phylogenetic analysis. However, in pan-genome phylogenetic analysis, the majority of the toxigenic strains were in cluster B (75.9%) whilst non-toxigenic resided in cluster A (63.6%). Further statistical analysis using Pearson’s Chi square test showed that there is a significant difference between cluster A and B with A consisting of non-toxigenic strains and vice versa at *p *= 0.001. The majority of Malaysia’s toxigenic isolates (85.2%) were clustered in B except for C110, C319, C517 and RZ358. These four isolates as well as toxigenic strains: TH510, TH1526 from India; CD1791, CD2173, CD72, CD2225, CD5052, CD4728 from Belarus; CD31A from Brazil along with NCTC13129 and NCTC5011 from United Kingdom, were scattered among the non-toxin bearing isolates. Among them, 3 out of 4 Malaysia’s toxigenic isolates (C110, C319, C517), except RZ358, claded with those from Belarus and United Kingdom in cluster A. These observations showed that there is a unique and close relationship between these non-toxigenic and toxigenic strains. Therefore, there is a possibility that the tox may not be the cause of the pathogenicity which may bear to the ineffectiveness of the toxoid vaccine. The rising awareness of the other virulence factors besides toxin has brought to the investigations on iron acquisition system, resistance mechanism, and pathogenicity islands [17, 20, 21].

The overall distribution of core genome and pan-genome SNP-based phylogenetic tree was different. The pan-genome SNP analysis is able to detect slight changes in genetically-close organism especially those in the accessory genomes, therefore further discriminate the strains with similar core genome. This could be due to the regrouping of the strains as a result of the SNP changes in accessory genomes compared to the conserved core genome. A similar observation was also depicted by Sangal et al. showing discrepancy in the clustering and degree of variation using the same set of strains in core vs accessory genome and proteome analysis [17]. A marked difference was noted when a large cluster of toxigenic strains were shifted to cluster B and both BH8 and CD31A from Brazil to cluster A in pan-genome SNP phylogenetic tree. The pan-genome SNP analysis has also brought Malaysia’s strains: RZ632 and RZ356 to be closer to Africa’s strains. It is also interesting to see that a number of recent outbreak strains from Malaysia, India and Africa in year 2016 were grouped closely to each other within cluster B. The clustering of the strains by both SNP analysis were slightly different with the core genome sequence alignment generated phylogenetic tree as reported by Hong et al. and Trost et al. [8, 20]. The intra-clustering within a clade may not be altered when genetically distinct species is introduced. However, in our study, the introduction of Belarus strains showed a high relatedness with Malaysia’s strains leading to the recalculation of the genetic difference and restructuring of the cluster.

PW8 (toxoid vaccine) is used as the reference and indicator for molecular analysis of *tox* and *DtxR* genes. All the local strains’ *tox* gene were aligned and compared against PW8 using Clustal Omega. One or two points mutation were detected at nucleotide level in *tox* but the amino acid sequences were in perfect sequence identity with PW8 except for RZ319 and RZ597 which presented a non-synonymous amino acid change by the substitution of histidine to tyrosine at position 24 (H24Y) with no deleterious effect as predicted by PROVEAN [18, 19]. This observation showed that Malaysia’s strains produce single antigenic type of toxin similar to the toxoid.

Genetic variations in the composition of *DtxR* might influence the *tox* gene expression and the virulence of *C. diphtheriae* [3, 4]. The analysis on local strains by comparing to PW8, showed that all except four *C. diphtheriae* strains, C110, C517, C319 and C113, had non-synonymous amino acid change in DtxR. Two non-synonymous SNPs: alanine to valine (A147V) and leucine to isoleucine (L214I) at position 147 and 214 respectively were located in C110, C517 and C319, all in cluster A. This observation is in concordance with a report shown by Nakao et al. who reported most amino acid substitution occurs in the carboxyl-terminal half of *DtxR* and both the amino acid substitution, A147 and L214I were observed in Russia and Ukraine strains [3]. However, a different observation in our isolate was the amino acid substitution at position 150, changing threonine to asparagine (T150N) of C113. However, all of them were predicted to be neutral by PROVEAN [18, 19].

Our analysis provides a general overview on the Malaysia’s *C. diphtheriae* isolates and the difference in genetic relatedness caused by the accessory genomes at a glance. Pan-genome SNP analysis allows a more rapid and efficient genetic relatedness observation using SNP variation especially in outbreak study to discriminate variations in core genome and accessory genome between genetically similar species [15, 16]. A further insight into the variability in the accessory genome between the closely related toxigenic and non-toxigenic local strains, for instance, RZ358, will be required to understand the acquired pathogenicity other than toxin such as the presence of functional genomic islands [17, 20, 21]. Our current analysis has significantly divided the toxigenic and non-toxigenic strain into two clusters, focusing mainly on local isolates. The observation might differ if more toxin-bearing clones with non-toxin related pathogenicity were introduced in the future.

In conclusion, over the years, sporadic diphtheria cases in Malaysia were shown to bear diverse strains. Based on the pan-genome SNP analysis, it is possible that the *C. diphtheriae* strains isolated in Malaysia could be of Belarus, Africa and India origin or vice versa based on the shared SNPs. However, the majority of the strains isolated in the year 2016 outbreak were clustered with strains isolated from as early as year 1986 indicating the presence of a persistent local strain in the population for decades. The non-toxigenic and toxigenic strains can also be clustered in A and B with regards to the toxin status. All the Malaysia clinical isolates produced single antigenic type of diphtheria toxin, similar to PW8. Given the well-conserved amino acid composition of toxin and DtxR of these local isolates compared to PW8, the alteration in the efficacy of the currently used toxoid vaccine would be unlikely.

## Limitations

The investigation on the specific type of accessory genome would be useful to understand the connection between toxigenic and non-toxigenic *Corynebacterium diphtheriae* strains in Malaysia. Most of the local *C. diphtheriae* isolated are toxigenic strains and only one non-toxigenic strain is available for analysis.

## Abbreviations

*C. diphtheriae*: *Corynebacterium diphtheriae*
DtxR: diphtheria toxin repressor
*Tox*: toxin gene
SNP: single nucleotide polymorphism

## References

[1] World Health Organization. Diphtheria reported cases. Last update: 6 September 2017. http://apps.who.int/immunization_monitoring/globalsummary/timeseries/tsincidencediphtheria.html. Accessed 29 Nov 2017.

[2] Hadfield TL, McEvoy P, Polotsky Y, Tzinserling VA, Yakovlev AA (2000). The pathology of diphtheria. J Infect Dis..

[3] Nakao H, Mazurova IK, Glushkevich T, Popovic T (1997). Analysis of heterogeneity of *Corynebacterium diphtheriae* toxin gene, *tox*, and its regulatory element, DtxR by direct sequencing. Res Microbial.

[4] Boyd JM, Hall KC, Murphy JR (1992). DNA sequences and characterization of *DtxR* alleles from *Corynebacterium diphtheriae* PW8(−), 1030(−), and C7hm723(−). J Bacteriol.

[5] World Health Organisation. Diphtheria global annual reported cases and DTP3 coverage, 1980–2016. Data as of 19 July 2017. http://www.who.int/immunization/monitoring_surveillance/burden/diphtheria/en/. Accessed 29 Nov 2017.

[6] Ahmad N, Hii SYF, Mohd Khalid MKN, Abd Wahab MA, Hashim R, Tang SN, Liow YL, Hamzah N, Dahalan NA, Seradja V (2017). First draft genome sequences of malaysian clinical isolates of *Corynebacterium diphtheriae*. Genome Announc..

[7] Engler KH, Glushkevich T, Mazurova IK, George RC, Efstratiou A (1997). A modified Elek test for detection of toxigenic corynebacteria in the diagnostic laboratory. J Clin Microbiol.

[8] Hong KW, Asmah Hani AW, Nurul Aina Murni CA, Pusparani RR, Chong CK, Verasahib K, Yusoff WN, Noordin NM, Tee KK, Yin WF, Yu CY, Ang GY, Chan KG (2017). Comparative genomic and phylogenetic analysis of a toxigenic clinical isolate of *Corynebacterium diphtheriae* strain B-D-16-78 from Malaysia. Infect Genet Evol..

[9] Plessis MD, Wolter N, Allam M, Gouveia LD, Moosa F, Ntshoe G, Blumberg L, Cohen C, Smith M, Mutevedzi P, Thomas J, Horne V, Moodley P, Archary M, Mahabeer Y, Mahomed S, Kuhn W, Mlisana K, McCarthy K, Gottberg AV (2017). Molecular characterization of *Corynebacterium diphtheriae* outbreak isolates, South Africa, March–June 2015. Emerg Infect Dis..

[10] Veeraraghavan B, Anandan S, Sekar SKR, Gopi R, Ragupathi NKD, Ramesh S, Verghese P, Korulla S, Mathai S, Sangal L, Joshi S (2016). First report on the draft genome sequences of *Corynebacterium diphtheriae* Isolates from India. Genome Announc..

[11] Grosse-Kock S, Kolodkina V, Schwalbe EC, Burkovski JBA, Hoskisson PA, Brisse B, Smith D, Sutcliffe IC, Titov L, Sangal V (2017). Genomic analysis of endemic clones of toxigenic and non-toxigenic *Corynebacterium diphtheriae* in Belarus during and after the major epidemic in 1990s. BMC Genomics..

[12] Gardner SN, Hall BG (2013). When whole-genome alignments just won’t work; kSNP v2 software for alignment-free SNP discovery and phylogenetics of hundreds of microbial genomes. PLoS ONE.

[13] Figtree v1.4.3. http://tree.bio.ed.ac.uk/software/figtree/.

[14] Sievers F, Wilm A, Dineen DG, Bibson TJ, Karplus K, Li W, McWilliam H, Remmert M, Söding J, Thompson JD, Higgins DG (2011). Fast, scalable generation of high-quality protein multiple sequence alignments using Clustal Omega. Mol Syst Biol..

[15] Medini D, Donati C, Tettelin H, Masignani V, Rappuoli R (2005). The microbial pan-genome. Curr Opin Genet Dev.

[16] Dangel A, Berger A, Konrad R, Bischoff H, Sing A (2018). Geographically diverse clusters of nontoxigenic *Corynebacterium diphtheriae* infection, Germany, 2016–2017. Emerg Infect Dis..

[17] Sangal V, Blom J, Sutcliffe IC, Hunolstein CV, Burkovski A, Hokisson PA (2015). Adherence and invasive properties of *Corynebacterium diphtheriae* strains correlates with the predicted membrane-associated and secreted proteome. BMC Genomics..

[18] Ng PC, Henikoff S (2006). Predicting the effects of amino acid substitutions on protein function. Annu Rev Genomics Hum Genet.

[19] Choi Y, Sims GE, Murphy S, Miller JR, Chan AP (2012). Predicting the functional effect of amino acid substitutions and indels. PLoS ONE.

[20] Trost E, Blom J, Soares SDC, Huang IH, Al-Dilaimi A, Schröder J, Jaenicke S, Dorella FA, Rocha FS, Miyoshi A, Azevedo V, Schneider MP, Silva A, Camello TC, Sabbadini PC, Santos CS, Santos LS, Hirata R, Mattos-Guaraldi AL, Efstratiou A, Schmitt MP, Hung TT, Tauch A (2012). Pangenomic study of *Corynebacterium diphtheriae* that provides insights into the genomic diversity of pathogenic isolates from cases of classical diphtheria, endocarditis and pneumonia. J Bacteriol.

[21] Cerdeño-Tárraga AM, Efstratiou A, Dover LG, Holden MTG, Pallen M, Bentley SD, Besra GS, Churcher C, James KD, Zoysa AD, Chillingworth T, Cronin A, Dowd L, Feltwell T, Hamlin N, Holroyd S, Jagels K, Moule S, Quail MA, Rabbinowitsch A, Rutherford KM, Thomson NR, Unwin L, Whitehead S, Barrell BG, Parkhill J (2003). The complete genome sequence and analysis of *Corynebacterium diphtheriae* NCTC13129. Nucleic Acids Res.

